# Implications of stress for animal welfare and animal-based research^†^

**DOI:** 10.1177/00236772251396310

**Published:** 2025-12-14

**Authors:** Sarah E. Wolfensohn, Vera Baumans, F. Josef van der Staay, Saskia S. Arndt

**Affiliations:** 1 University of Surrey, Faculty of Health and Medical Sciences, School of Veterinary Medicine, Guildford, Surrey, UK; 2 Division Animals in Science and Society, Department of Population Health Sciences, Faculty of Veterinary Medicine, Utrecht University, Utrecht, the Netherlands; 3 Behavior & Welfare Group (Formerly Emotion & Cognition Group), Division of Farm Animal Health, Department of Population Health Sciences, Faculty of Veterinary Medicine, University Utrecht, Utrecht, the Netherlands

**Keywords:** Adaptive behaviour, homeostasis, welfare assessment tools

## Abstract

Better animal welfare is essential for better animal-based science. Poor welfare induces stress (and vice versa), which in turn can affect or even severely confound research results. To ensure the validity of scientific results, those who work with laboratory animals must take responsibility for their care and welfare. A harm–benefit analysis can be used to weigh the welfare and quality of life of animals in scientific studies against the resulting benefits to other animals or humans, provided that animal welfare can be validly assessed. This review considers the identification and characterisation of stress by physiological, hormonal, immunological and behavioural measures and by assessment of the physical condition of the animal. It addresses controllability, predictability, chronicity, duration and severity, and the intrinsic and extrinsic factors that modulate animal behaviour and coping mechanisms in response to stress. EU Directive 2010/63 requires procedures to be classified as mild, moderate or severe. However, as long as some researchers use purely subjective assessments without a supporting structure and reference scales, or no assessment method at all, these terms may have limited practical value. The challenge is to understand the state of the animal from the information available. Welfare assessment tools can be used to demonstrate the true impact of research procedures and their refinement to protect animal welfare. If any doubt exists about the harm–benefit evaluation of the experiment then the welfare of the animal should take priority.

## Implications du stress pour le bien-être animal et la recherche basée sur les animaux

Résumé

Un meilleur bien-être animal est essentiel pour assurer une meilleure science basée sur les animaux. Un mauvais bien-être induit un stress (et *vice versa*), qui à son tour peut affecter voire fausser gravement les résultats de la recherche. Pour garantir la validité des résultats scientifiques, les personnes qui travaillent avec des animaux de laboratoire doivent assumer la responsabilité de leurs soins et de leur bien-être.

Une analyse dommages-avantages peut être utilisée pour évaluer le bien-être et la qualité de vie des animaux dans les études scientifiques par rapport aux avantages qui en résultent pour d'autres animaux ou humains, à condition que le bien-être animal puisse être valablement évalué. Le présent examen examine l'identification et la caractérisation du stress par des mesures physiologiques, hormonales, immunologiques et comportementales et par l'évaluation de la condition physique de l'animal. Il aborde la contrôlabilité, la prévisibilité, la chronicité, la durée et la gravité, ainsi que les facteurs intrinsèques et extrinsèques qui modulent le comportement animal et les mécanismes d’adaptation en réponse au stress.

La directive 2010/63 de l'UE exige que les procédures soient classées comme légères, modérées ou sévères. Tant que certains chercheurs utilisent des évaluations purement subjectives sans structure de soutien ni échelles de référence, ou sans méthode d'évaluation, ces termes peuvent toutefois ne revêtir qu’une valeur pratique limitée. Le défi consiste à comprendre l'état de l'animal à partir des informations disponibles. Les outils d'évaluation du bien-être peuvent être utilisés pour démontrer l'impact réel des interventions de recherche et leur raffinement pour protéger le bien-être animal. En cas de doute quant à l'évaluation des risques et des avantages de l'expérience, le bien-être de l'animal devrait être prioritaire.

## Auswirkungen von Stress auf das Wohlergehen von Tieren und die Versuchstier-Forschung

Optimaler Tierschutz ist für eine bessere Tierforschung unerlässlich. Schlechte Haltungsbedingungen verursachen Stress (und umgekehrt), was wiederum die Forschungsergebnisse beeinflussen bzw. sogar stark verfälschen kann. Um die Validität wissenschaftlicher Ergebnisse zu gewährleisten, müssen mit Labortieren befasste Personen Verantwortung für deren Pflege und Wohlergehen übernehmen.

Eine Nutzen-Risiko-Analyse kann dazu dienen, das Wohlergehen und die Lebensqualität von Tieren in wissenschaftlichen Studien gegen den daraus resultierenden Nutzen für andere Tiere oder Menschen abzuwägen, vorausgesetzt, das Wohlergehen der Tiere kann valide bewertet werden. Die vorliegende Untersuchung befasst sich mit der Identifizierung und Charakterisierung von Stress anhand physiologischer, hormoneller, immunologischer und verhaltensbezogener Messungen sowie anhand der Beurteilung des körperlichen Zustands des Tieres. Sie berücksichtigt Kontrollierbarkeit, Vorhersagbarkeit, Chronizität, Dauer und Schweregrad sowie die intrinsischen und extrinsischen Faktoren, die das Verhalten der Tiere und ihre Bewältigungsmechanismen als Reaktion auf Stress modulieren.

Die EU-Richtlinie 2010/63 fordert, den Schweregrad der Verfahren als leicht, mittel oder schwer einzustufen. Solange jedoch einige Forscher rein subjektive Bewertungen ohne unterstützende Struktur und Referenzskalen oder gar keine Bewertungsmethode verwenden, sind diese Begriffe vermutlich nur von begrenztem praktischen Wert. Die Herausforderung besteht darin, den Zustand des Tieres anhand der verfügbaren Informationen zu verstehen. Mit Hilfe von Instrumenten zur Bewertung des Wohlergehens lassen sich die tatsächlichen Auswirkungen von Forschungsverfahren und deren Verfeinerung zum Schutz des Wohlergehens der Tiere aufzeigen. Bestehen Zweifel hinsichtlich der Nutzen-Risiko-Bewertung des Experiments, muss das Wohlergehen des Tieres Vorrang haben.

## Implicaciones del estrés para el bienestar animal y la investigación basada en animales

Resumen

Un mejor bienestar animal es fundamental para mejorar la ciencia basada en animales. Un bienestar deficiente provoca estrés (y viceversa), lo cual puede afectar, o incluso sesgar de manera significativa, los resultados de la investigación. Para garantizar la validez de los resultados científicos, quienes trabajan con animales de laboratorio deben asumir la responsabilidad de su cuidado y bienestar.

Se puede emplear un análisis de daños y beneficios para ponderar el bienestar y la calidad de vida de los animales utilizados en estudios científicos frente a los beneficios resultantes para otros animales o seres humanos, siempre que el bienestar animal pueda evaluarse de manera válida. Esta revisión aborda la identificación y caracterización del estrés mediante medidas fisiológicas, hormonales, inmunológicas y conductuales, así como mediante la evaluación del estado físico del animal. Asimismo, analiza la capacidad de control, la previsibilidad, la cronicidad, la duración y la gravedad, así como los factores intrínsecos y extrínsecos que modulan el comportamiento animal y los mecanismos de afrontamiento en respuesta al estrés.

La Directiva 2010/63 de la UE exige que los procedimientos se clasifiquen como leves, moderados o graves. Sin embargo, mientras algunos investigadores empleen evaluaciones puramente subjetivas sin una estructura de apoyo y escalas de referencia, o directamente sin ningún método de evaluación, estos términos pueden tener un valor práctico limitado. El reto consiste en comprender el estado del animal a partir de la información disponible. Las herramientas de evaluación del bienestar pueden utilizarse para demostrar el impacto real de los procedimientos de investigación y su refinamiento con el fin de proteger el bienestar animal. Si existe alguna duda en la evaluación del balance daño-beneficio del experimento, entonces deberá prevalecer el bienestar del animal.

## Introduction

The nature of the relationship between humans and animals has changed considerably during the process of domestication: animals have been bred for the provision of products of animal origin, or they have become pets that live with humans as companions. A third group of animals, used for research, has also been established: laboratory animals.

Most are descended from domesticated groups such as pet mice and rats, rabbits, cats and dogs, or farmed animals such as chickens and pigs.

As a result of being bred specifically for experimental purposes or for intensive farming, these animals often have problems adapting to their housing conditions.^1^ Modern housing systems for farm and laboratory animals often result in behavioural and physiological abnormalities because they do not meet the minimum requirements to satisfy the animals’ needs, let alone what they actually want,^2^ promoting positive animal welfare experiences.^3–5^

Animal welfare legislation imposes a ‘duty of care’ to provide for the basic needs of animals, which include a suitable environment, appropriate food, the opportunity to exhibit normal behaviours, to be housed with (or separated from, as applicable) other animals, and to be protected from pain, injury, suffering and disease. Various codes of practice give practical advice on how to ensure that these welfare needs are met.^6^ However Yeates and Main^7^ raised the question ‘what use is there in satisfying an animal’s vital needs, if the life the animal then lives is devoid of any enjoyment?’ (p. 298), since enjoyment will only be achieved by fulfilling wants, not just needs. It should be noted, however, that constantly satisfying animal desires may have negative long-term effects, such as obesity in several dog breeds,^8^ when given free access to food.

From a legal as well as from an ethical perspective, it is imperative to safeguard and increase welfare and minimise the suffering of animals: better welfare is inherent to better science. As stated by Poole,^9^ ‘to avoid confounding variables, experimental animals should have both normal physiology and behaviour’ (p. 117). Implementation of European legislation demands a detailed and thorough knowledge of the biology of the various animal species being used in experiments. Scientists need to gain indirect information by studying the animal’s dynamic interaction with its environment and observing its behaviours, such as facial expressions, which can reflect stress or pain.^10^

This article discusses the importance of stress detection and control in laboratory animal research, as stress not only affects animal welfare, but can also modulate or even invalidate study results. The animal should reach a welfare state that it perceives as positive, and the science must take account of the adaptation mechanisms, the development of stress pathology, and the consequences for the animals’ welfare. The modern concepts of welfare are much more than simply the absence of suffering and negative impacts; they require the presence of positive experiences as well.^4^ As Wathes et al. stated in 2009, it is about ensuring that the animal has a life worth living or, even better, a good life.^11^ This standard should be applied to animals used in laboratories to ensure that they can be valid subjects for research and to protect their health and welfare. Judgement of the quality of life of laboratory animals against the benefits to other animals, or humans, is the basis of the harm–benefit analysis. It has been argued that the animal’s quality of life should be used for decision-making based on an objective assessment of quality of life.^12,13^

## Animal welfare definitions, concepts and frameworks

It is important to be clear about which concept of welfare is being used, since definitions vary.^14^ Unfortunately, there is no consensus on one definition or concept of animal welfare, but the two most prevalent concepts are currently the Five Freedoms and the Five Domains model.

### The Five Freedoms

One of the first concepts, ‘The Five Freedoms’, formalised in 1979 by the UK Farm Animal Welfare Council, was based on the seminal work of the Brambell Committee.^15^ This concept calls for freedom from 1) thirst, hunger, malnutrition, 2) suffering, 3) pain, injury, disease, 4) the freedom to express normal behaviour, and 5) freedom from fear and distress^16^ in order to safeguard welfare. Interestingly, although the Five Freedoms were developed in the context of farm animal welfare, they have since been used as a guide for assessing the welfare of animals kept in a variety of contexts, including research laboratories.^17–19^ This concept requires the absence of negative states (except the fourth freedom), but misses the point that welfare also requires the presence of positive states.^20^

### The Five Domains model

In 1994, Mellor and Reid^21^ introduced a first version of the Five Domains model, which includes five interacting physical/functional domains: 1) nutrition, 2) environment, 3) health, 4) behaviour, and 5) mental state. This concept seeks to assess the impact of the physical and social environment on the affective state of an animal. Taking a predominantly physiological orientation, the model was structured to first evaluate ‘particular physical/functional disruptions and imbalances, as well as restrictions on behavioural expression, and then to identify the specific negative affects each disruption, imbalance or restriction would be likely to generate’ (p. 8).^22^

### A dynamic concept of animal welfare

A welfare concept should take the animal’s ability to cope with environmental challenges, the importance of negative and positive emotions, into account. That is why the dynamic concept of animal welfare has been introduced (note, the following has been slightly modified from Arndt et al.^4^):

An individual is likely to be in a positive welfare state if he or she is mentally and physically capable and has the ability and opportunity to respond appropriately to sporadic or sustained appetitive and adverse internal and external stimuli, events and conditions. Appropriate responses are elements of an animal’s normal behaviour. They enable the animal to cope with and adapt to the demands of the (prevailing) environmental circumstances and to reach a state that he or she perceives as positive, i.e. that evokes positive emotions.^23^

Animals are assumed to be highly motivated to perform natural behaviours, which may help to reduce or avoid the types of stress that lead to a deterioration in the animal’s adaptive capacity.^4^ Normal behaviour is crucial in social interactions, as it makes an animal’s actions and interactions with other animals (including members of other species) predictable and unambiguous.^24,25^ Predictability reduces stress, whereas unpredictability induces stress.

Intrinsic and extrinsic adverse factors may sporadically or constitutionally affect welfare. Sporadic effects are short-lived and cause an immediate stress response, whereas constitutional effects are long-lived and are often caused by the animal’s intrinsic condition, such as gene mutations, the effects of infectious or toxic agents, as well as age-related deficits, lack of socialisation,^26^ disrupted hormonal balance after neutering,^27,28^ and suffering from a chronic disease.

**Figure 1.**
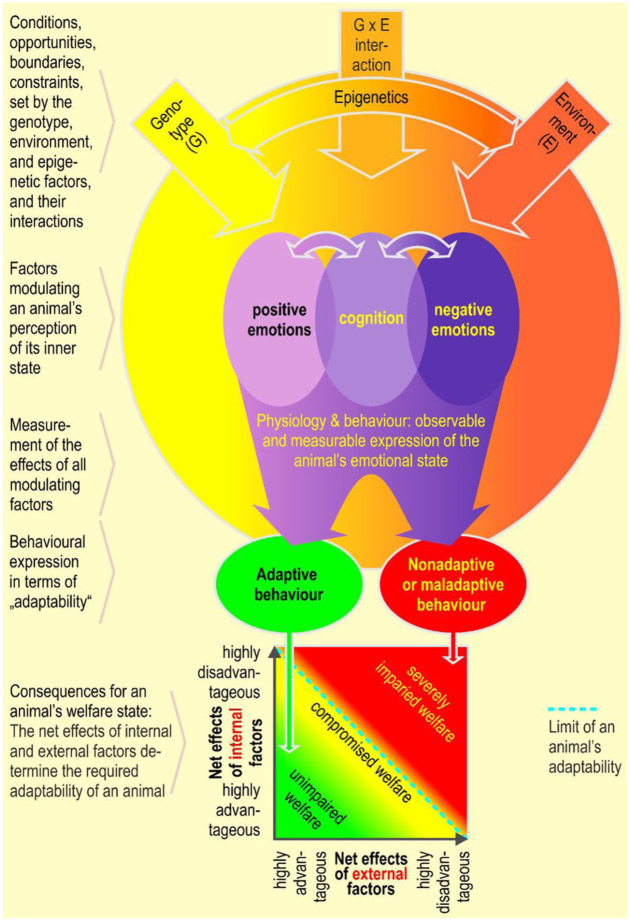
An animal’s emotions and cognitive performance are deployed and expressed within the conditions and constraints set by the genotype, environment and their interactions. The double-headed arrows indicate that emotions modulate cognitive processes and vice versa.

If an animal can easily adapt to and cope with the effects of these adverse factors, the impact on its welfare may be transient. However, if significant adaptive capacity is required, that is, if the adverse factors exceed the limits of the individual’s mental, cognitive and physical capacity and ability to respond appropriately, then the welfare of an animal is likely to be seriously compromised as it will not be able to deal with the situation within its normal behavioural repertoire (see Figure 1). Factors such as inadequate housing and management practices will reduce the animal’s coping ability. Pain caused by injections or surgery are examples of sporadic intrinsic factors, whereas a visit by the veterinarian or the transient effects of regrouping social animals are examples of the adverse impact of sporadic extrinsic factors.

The strategies to improve animal welfare will differ, depending on whether they are caused by the actions of adverse, stress-inducing sporadic or constitutional factors and whether these factors are intrinsic or extrinsic. Changes in extrinsic (environmental) conditions can have an immediate effect on an animal’s behaviour and welfare, whereas changes in some intrinsic factors, such as changes in the genome, can only be achieved over a number of successive generations and do not have an immediate effect on an animal’s compromised welfare.

## Factors (potentially) influencing behaviour and experimental results

Stress may modulate behaviour and many physiological and hormonal parameters. The behaviour of an animal itself is an intrinsic factor (controlled by the animal’s own bodily and mental processes), whereas the behaviour of other animals (family/group/herd members) may be seen as an extrinsic factor that is part of the animal’s physical environment and will affect and modulate its behaviour.

### Intrinsic factors (animal related)

Intrinsic factors are of physiological or psychological origin. Stress has been described as reflecting the subject’s perception and interpretations of its internal physical sensations.^29^ In animals, these factors are not directly measurable, as animals are unable to report the experience of stress, and must therefore be deduced from the animals’ behaviour when coping with the stressor(s), usually behaviour that leads to the avoiding of, or escaping from, the stressor.^30,31^ The emotional expression of stress has been described by components such as changes in the peripheral nervous system, the hormonal system, and overt behaviours, including facial expressions.^32^

Physical and cognitive abilities and capacities, state of health, intrinsic bodily sensations, intrinsic bodily cues, function of endocrine organs, and a sense of controllability of the adverse situation causing stress have all been described as intrinsic factors.^30^

According to Averill,^33^ three types of control may be distinguished, namely behavioural-, cognitive-, and decisional control. Behavioural control refers to the animal’s direct action on the environment, cognitive control refers to the interpretation of the events that cause stress, and decisional control refers to the availability of choice alternatives for dealing with the stressor.

### Extrinsic factors (environment related)

The extrinsic factors are part of the physical and social environment. The behaviour of an animal is strongly linked to certain specific locations and conditions of its physical and social environment, as exemplified in Table 1. Many studies have shown that behaviour has a strong genetic basis, as evolution has eliminated the less genetically adapted individuals. This form of selection is the basis of species-specific behaviour, as well as the species-specific morphology and physiology of animals, including that of present-day experimental animals. In their original, natural habitat, animals are characterised according to the adequacy of their morphology, physiology and behaviour. The regulatory range of homeostatic mechanisms is therefore geared up for and restricted to the range of environments in which they have evolved. Therefore, the adequacy of homeostatic mechanisms may fail, should changes in the environment require completely different characteristics of these mechanisms.

**Table 1.** Examples of intrinsic and extrinsic factors that, depending on how they manifest, may trigger stress.

Intrinsic factors (animal related)	Extrinsic factors (environment related)
Physical and cognitive abilities and capacities	Physical and social environmental conditions
Health state	Availability of food, water, shelter
Function of endocrine organs	Crowding conditions
Sense of controllability of the adverse situation	Hierarchy in groups (stable/unstable)
Predictability of the adverse situation	
Stage in development (ontogeny, ageing)	Mother–child bond
Emotional state	Quality of food, water, shelter

During the process of domestication, experimental animals have acquired new specific characteristics. This does not mean, however, that predomestication species-specific behaviour has disappeared completely.^34^ Behaviour that is essential for survival (feeding, nest building and social behaviour such as offensive, defensive, sexual and parental behaviour) is strongly genetically determined. These behaviours will be present in offspring even if selection is not in their favour. Species-specific behaviours are highly resistant to change, and they will be performed even if the environment does not allow their full expression. When two strains of laboratory rats, being bred in the lab for many generations, were released in a semi-natural habitat outside the lab, they were perfectly able to cope with the new environmental conditions,^35^ an observation that may apply to nearly all domesticated species.^34^

Selection by the environment is an important factor in the phylogeny of adaptive behaviour. Animals are, however, not fully dependent upon genetically programmed behaviour. Cognitive processes help the animal to adapt to changing living conditions.

The relationship between animals and humans has the potential to act as a stabilising or disruptive factor. For instance, in pig husbandry, a human factor appears to be involved in the growth^36–38^ and reproduction of the animals. This issue is the subject of ongoing research. Experimental evidence supports the notion that the quality of the human–animal interaction may affect the health of the animal^39^ and, therefore, the quality and reliability of animal experimental results.

For example, the environment during the housing and testing of an animal in the research laboratory can potentially affect the results of a study.^40^ Environmental factors include, for example, climatic conditions, feeding, housing (e.g. cage size, cage enrichment, number of animals per cage), and caretaker handling of animals. In the case of re-use of an animal or testing in a battery of tests within the same study, previous experience has been described as potentially influencing behaviour in the next study/test.^41^

Many animals used in studies spend a considerable proportion of their life at the breeder’s facilities where housing conditions and animal care routines may be different from the research laboratory. The conditions in the breeder’s facilities are often unknown to the researcher^42^ and are not under experimental control. Stress during the pre-experimental phase may exert a lasting effect on the results of a study and may be considerable, but go unnoticed. Maternal care, weaning, and mixing at the breeder facility are all likely to affect experimental results, as are factors such as transportation from the breeder to the research laboratory, construction noise from building modifications, and the effects of previous experiments on the same animal.^41,43,44^ The confounding effects of changing the experimenter during testing and changing the order of test animals housed in the same cage have also been discussed.^45,46^

## Stress-regulating systems and functional implications

### Homeostasis/allostasis

Homeostasis is a useful concept when discussing stress, adaptation and animal welfare. This implies that aspects of both the internal milieu (such as body temperature, blood glucose, water content of the body) and those of the environment (like position within a social group, etc.) will be kept at a constant or at least a predictable level for a certain period of time. An animal can only maintain homeostasis when it can compare its actual situation with the norm for certain internal or external factors, and when it has the behavioural and physiological capacity and ability to carry these norms into effect, that is, to successfully adapt to these conditions.

**Figure 2.**
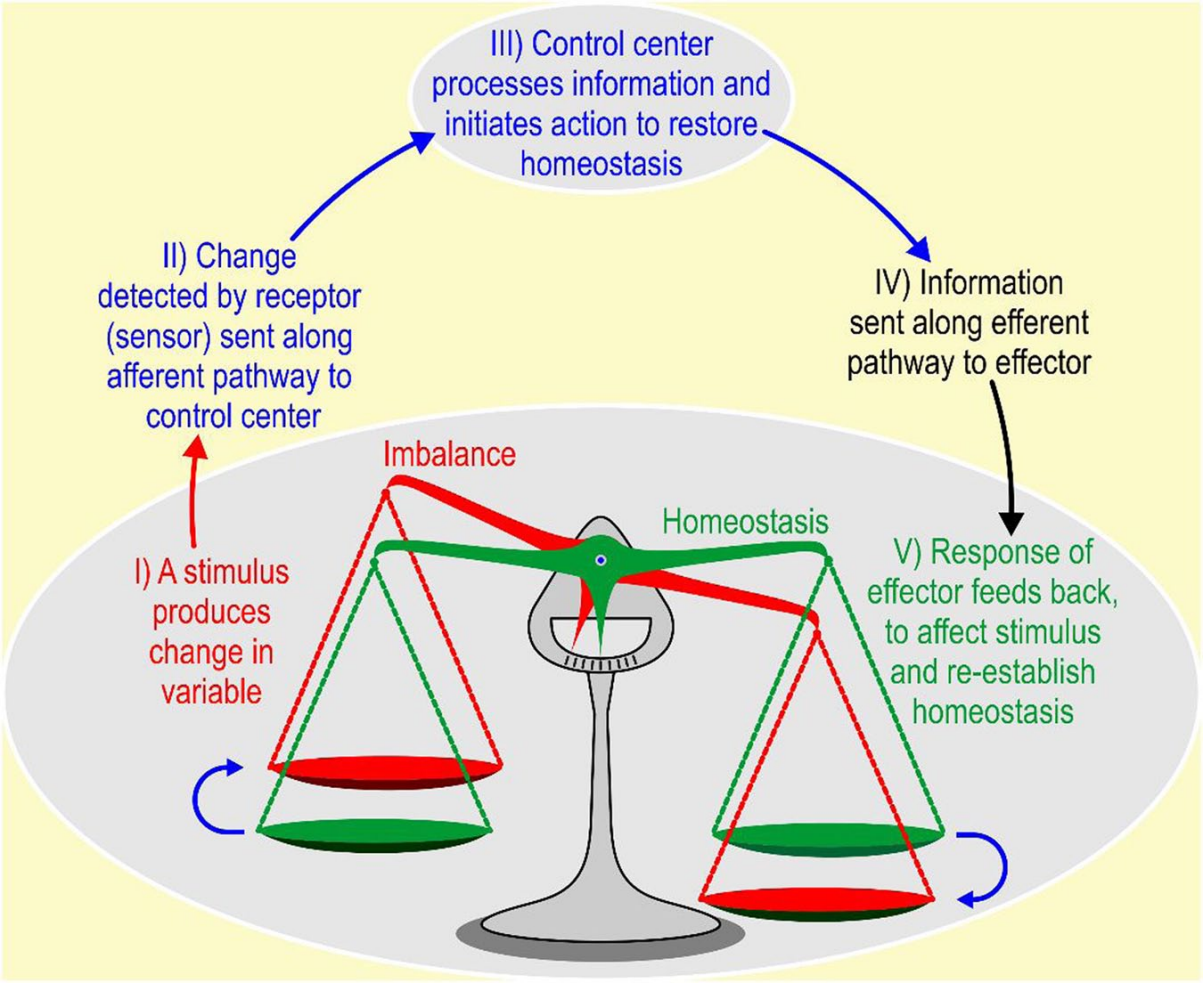
Principle of a homeostatic mechanism. Stimulus-induced homeostatic imbalance (*I*) is detected (*II*), processed and leads to the initiation of restoration (*III*) through an efferent pathway (*IV*), affecting the stimulus and therewith re-establishing homeostasis (*V*).

A stress response begins with the central nervous system perceiving a potential threat to homeostasis. This leads to a biological response (see Figure 2). If the animal is unable to maintain homeostasis, stress might develop until the animal adapts to this situation and re-establishes homeostasis. If the animal is not able to adapt to a certain situation, stress might change into distress.

Situations that extend the animal beyond its homeostatic capacities may result in allostasis, the ability of the body to achieve an alternative form of stasis. The long-term consequences of exposure to a stressor might then result in an allostatic load – the price of adapting to long-term stress.^47^

### The autonomic nervous system

The autonomic nervous system has two major subdivisions: the sympathetic and the parasympathetic. With only a few exceptions, all organs are innervated by both systems. The sympathetic and parasympathetic branches of the autonomic nervous system are active in reaction to a stressor.^48^ The sympathetic (fight-or-flight) and parasympathetic (relaxation) nervous systems are generally balanced in their activity. However, the two can be activated separately by specific stressors.^33^

### The neuroendocrine system

The hypothalamus–pituitary–adrenocortical (HPA) axis is classically involved in the neuroendocrine stress response. Selye^49^ was the first to demonstrate that a wide variety of stressors such as heat, cold or tissue damage are able to activate this system. This led to the formulation of the general adaptation syndrome (GAS) theory. Three different phases are distinguishable when an animal is chronically exposed to stressors. The first is the alarm phase, which is the direct physiological reaction to a stressor. After a while this reaction modifies into the second phase, that of resistance, in which the animal adapts its physiology to the continuous presence of the stressor. The increased excretion of adrenocortical hormones (mainly cortisol and corticosterone) may result in a hypertrophy of the adrenal cortex. In this situation, according to the Selyean stress concept, the animal has an enhanced resistance to the stressor. In the third phase, a state of exhaustion may occur, particularly when the animal is continuously exposed to quite severe stressors: the physiological capacity of the animal is not sufficient in meeting the environmental demands, and the animal will die of stomach ulcers, infections, and so forth. In case of extreme chronic stress, the GAS symptoms, as elaborated by Selye, are clearly distinguishable.^44,45,49,50^

**Figure 3.**
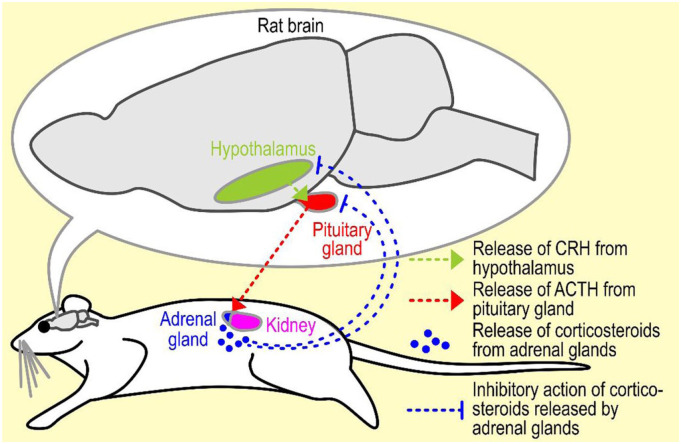
Schematic representation of the hypothalamic–pituitary–adrenal (HPA) axis in the rat. CRH: Corticotropin-releasing hormone; ACTH: adrenocorticotropic hormone.

Figure 3 depicts the HPA axis in the rat. ACTH (adrenocorticotropic hormone) is the pituitary hormone that activates the adrenal cortex in secreting corticosterone. This hormone is synthesised from a large precursor molecule known as pro-opiomelanocortin. A number of other stress hormones are derived from this precursor molecule. One of them is ß-endorphin, an endogenous opiate.

### Functional significance of physiological stress responses

Physiological parameters such as elevated plasma levels of catecholamines or corticosteroids are often used as indicators that an animal’s welfare is compromised or impaired, but these parameters are adaptive and necessary for the organism to cope with the present (stressful) conditions and to survive. The physiological changes of the organism in reaction to a stressor are important on two organisational levels. Both the autonomic nervous system and the different neuroendocrine systems affect peripheral organs such as the heart, blood vessels, and immune system, as they function to prepare the peripheral physiological processes such as blood pressure, blood distribution, metabolism and immunocompetence in producing an adequate behavioural and physiological stress response.

## Stress responses and ways to cope with stress

### Effects of stress on learning

The cognitive ability of an animal (see Figure 1) and its modulation by stress have been intensively studied. Central to the consideration of stress are its controllability and predictability. Acute stress is the state of an animal after a sudden decrease in the predictability and/or the controllability of relevant environmental changes, and chronic stress is the state of an animal that occurs when relevant environmental aspects have a low predictability and/or are not very controllable over a long period of time. While predictability is closely associated with Pavlovian (classical) conditioning, controllability is associated with operant conditioning.^25^

Classical conditioning allows the animal to predict certain events in its environment, for example, the arrival of the keeper may predict food. By means of operant conditioning the animal learns which behaviours will cause significant changes in its umwelt (i.e. the specific way organisms of a particular species experience the world based on their sensory and perceptual systems), for example, obtaining food or avoiding a rival. This implies that animals have expectations about future events. These expectations are not, however, always realised. When reality seems to be different from what the animal expected, it is possible to observe emotional expressions.^51^ Examples include tail wagging, piloerection, various facial expressions, vocalisations (barking) and changes in heart rate in dogs. However, an ideal environment should not be totally predictable and controllable since there is evidence that a certain degree of unpredictability is required to avoid the negative aspects of boredom.^24,52^

### Coping styles

The coping mechanisms available to an animal determine whether or not it can successfully control its social and physical environment. If the situation is perceived as threatening homeostasis, coping mechanisms will be activated, but the reaction will vary from individual to individual. A variety of coping styles can be observed in different animal species.

For example, in rats and mice two extremes of reactions have been distinguished^53^: the proactive coping style, characterised by attempts to actively control the environment, and the reactive coping style, characterised by a predominantly passive acceptance of the situation. Animals characterised by a proactive coping style react predominantly with a sympathetic response, whereas animals with a reactive coping style have a more reactive system and show parasympathetic stress responses.^53^ Due to the differential involvement of the autonomic nervous systems in various types of pathology, this may indicate an individual differentiation in susceptibility to stress pathology.

### Emotional repertoire of different species

The repertoire of emotions differs between species, that is, the basic set of emotions is species-specific. Panksepp and Watt^54^ list seeking, fear, rage, lust, care, panic/grief and play as basic emotions, but point out that there may be more, and others have compiled different lists.^55^ Basic emotions, such as fear, trigger a stress response that is impacted by extrinsic (i.e. physical environmental) events or intrinsic (i.e. physiological or psychological) factors. These stressors cause a change in the animal’s biological balance.^56^ For any strategy to reduce stress in animals and improve their welfare, the identification of these stressors, their controllability, predictability, chronicity, duration and severity is of paramount importance. All these factors may have a relevant effect and must be considered when interpreting and discussing the results of a study.

Within its genetic limits, ontogeny makes a major contribution to the adaptability of the organism. Both prenatal and postnatal factors have a significant influence on both behavioural and physiological characteristics at adolescence. In mammals the internal milieu of the mother will affect the development of the offspring from the moment of conception. For example, offspring from mothers who experienced stress during pregnancy or nursing will show different social behaviour in adulthood.^57^ Experience with conspecifics is also important in the development of adult social behaviour. The complexity and variability of the environment will greatly influence the development of exploratory behaviour and cognitive abilities, and determine the adult’s adaptability to environmental challenges.

## Measurements of stress and welfare

### Physiological measurements

Measurement of physiological stress has traditionally focused on monitoring autonomic responses, such as changes in heart rate, respiration rate, blood pressure and/or temperature.^58–60^ The effects of routine experimental procedures on physiological parameters have been found to differ in mice kept under different housing conditions.^61^

Whereas implantation of telemetry devices to measure cardiovascular parameters may increase and confound stress measures,^53^ the technology for these devices is developing rapidly. It is important to ensure that sampling for the sole purpose of measuring welfare does not contribute to the negative welfare status of the animal.^62–64^

### Hormonal measurements

Another traditional measurement of physiological stress has focused on the detection of HPA axis hormones such as the glucocorticoids cortisol and corticosterone in blood, faeces, urine, saliva, tears or nails/feathers/hair.^65,66^ The collection of faeces, urine or saliva samples for cortisol assay has been described as practically complex, particularly from group-housed animals on a forage substrate. Also, the interpretation of cortisol assay results is complicated by considerable individual variation,^67^ a natural circadian rhythm in cortisol levels,^68–70^ the fact that a cortisol response is associated with some non-stress stimuli, and that some stress responses may not involve elevated cortisol levels.^71^ These problems have contributed to a growing reluctance in the field to use cortisol concentration as a (sole) measure of stress.^66–68,71,72^

### Immunological measurements

Neutrophil activation assays or leukocyte cell counts can reflect the effect of stress on the immune response. The leukocyte activation test measures the degree to which blood can produce a further neutrophil response to an *in vitro* challenge. Animals undergoing stress will produce a significantly lower leukocyte response than animals that are not stressed.^73^

### Physical measurements: body condition and alopecia

Body weight, which must take into account age, development, sex and reproductive condition, and body condition scoring can all be used to assess the physical condition of the animal. Many captive animals show forms of pelage loss, which results from grooming or plucking behaviours directed at themselves or at other individuals. This behaviour appears to be stress-related. In primates, it is a pathological intensification of natural grooming behaviour, frequently with hair ingestion, and is a symptom of psychogenic maladjustment to a poor environment, which can be reduced using various types of environmental enrichment. Quantifying alopecia in primates has therefore been described as potentially useful for compromised welfare assessment.^74^ Such scoring systems may also prove useful in other species (e.g. for quantifying barbering in laboratory rodents).

### Behavioural measurements

Behavioural analysis is complex: it involves assessing a wide range of behaviours, depending on the species, and is best carried out using a species-specific ethogram.^75^ The aim of systematic behavioural observation is to ensure that animals are able to display their behavioural repertoires and to take steps to facilitate their expression by providing appropriate environmental enrichment.^76^

Conflict behaviour that occurs in acute stress situations is often characterised by its short duration and the intensity of this behaviour may be exaggerated. In a situation where such conflicts cannot be resolved, and are of a more permanent nature, chronic stress will occur. The original conflict behaviour may be transformed into deviant behaviour. The best-known pathological behaviours are stereotypies and behaviours that cause harm to the individual or to others.

Stereotypies are frequently observed in various captive species. They are characterised by a relatively simple and constant structure, are frequently repeated, appear to have no specific purpose, and the form and expression of stereotypies are characteristic of the individual. Stereotypies can be considered to be ritualisation of conflict behaviours. Studies of voles in small cages have shown that their stereotypies can be reduced by using the opioid antagonist naloxone.^77^ This suggests that the occurrence of stereotypy is somehow related to the release of endorphins from the central nervous system, which may shed light on its functional significance.

However, even if stereotypies may be biologically significant to the animal, their incidence indicates that the animals have been (or still are) in a state of chronic stress. Hence, housing conditions within which stereotypies develop, should be avoided.

For example, harmful behaviour has been observed in socially isolated monkeys, who may bite their fingers, and in mink on commercial fur farms, where their tails are often severely damaged by constant sucking.^78^ Although the cause of this abnormal behaviour is not always known, it can be concluded that it indicates serious deficiencies in housing and care.

### Preferences

Preference tests in which animals are offered a choice of conditions, such as housing systems, bedding material and food, can be applied to provide information on the relative importance of environmental factors.^79^ However, care must be taken when interpreting the obtained results in terms of welfare. An animal’s choice may be biased by previous experience, or it may lack insight into what choice is good for its own long-term welfare (e.g. uncontrolled feeding leading to overeating and obesity).^8^

### Combining a range of assessment parameters into one usable welfare indicator

The explicit inclusion of positive welfare outcomes rather than simply assessing the presence of negative outcomes is characteristic of most current animal welfare definitions.^20^ Using clinical judgement and experience can provide a first indication of welfare. However, these should be complemented by other validated assessment methods.

Creating a usable overall indicator by combining a number of assessment parameters is an important objective in providing a practical, objective and robust animal welfare assessment tool. The Animal Welfare Assessment Grid,^13,80–85^ for example, records physical health, psychological welfare, environmental comfort and procedural events, and it illustrates the temporal component of suffering and quality of life. Use of the tool allows demonstration that the research facility is adopting a culture of care, which is about improving animal welfare, the quality of scientific research, the care of animal staff and improves transparency for stakeholders and the public.

## Conclusions

EU Directive 2010/63^86^ requires that procedures are classified according to severity level as being mild, moderate or severe. However, these are subjective terms open to interpretation. Unfortunately, some researchers continue to use purely subjective assessments that are open to debate, or no method of assessment at all. Without a supporting structure and scales of reference, these terms are of limited operational value. The challenge is to decipher the state of the animal from the information available, and then define the stress level it is experiencing. If any doubt exists about the harm–benefit evaluation of the experiment, then the welfare of the animal must take priority, and the responsibility for this lies with the researcher.

In summary, it can be said that acute physiological stress responses have two functions: they organise the organism to cope behaviourally and physiologically with the challenge and, at the same time, they facilitate learning and memory processes that allow the animal to react more effectively to a similar stressor in the future.

Welfare assessment tools can be used to demonstrate the true implications of research procedures on the animals’ wellbeing and the positive effects of refinements (i.e. to demonstrate that their implementation is effective). All those who interact with laboratory animals must accept responsibility in delivering animal welfare and, in doing so, will help to ensure the validity of science.
